# Non-culprit plaque characteristics in acute coronary syndrome patients with raised hemoglobinA1c: an intravascular optical coherence tomography study

**DOI:** 10.1186/s12933-018-0729-5

**Published:** 2018-06-15

**Authors:** Shaotao Zhang, Jiannan Dai, Haibo Jia, Sining Hu, Hongwei Du, Ning Li, Yongpeng Zou, Yanan Zou, Shenhong Jing, Yan Wang, Rong Sun, Bo Yu

**Affiliations:** Department of Cardiology, The 2nd Affiliated Hospital of Harbin Medical University, The Key Laboratory of Myocardial Ischemia, Chinese Ministry of Education, Harbin, China

**Keywords:** Pre-diabetes, Raised hemoglobinA1c, Optical coherence tomography, Coronary artery disease

## Abstract

**Background:**

Raised hemoglobinA1c (HbA1c) is an indicator of pre-diabetes, which is associated with increased risk of coronary artery disease. However, the detailed morphological characteristics of non-culprit plaques in acute coronary syndrome (ACS) patients remain largely unknown.

**Methods:**

A total of 305 non-culprit plaques from 216 ACS patients were analyzed by intravascular optical coherence tomography. These patients were divided into three groups according to the serum glycosylated hemoglobin level: normal HbA1c (< 5.7%), pre-diabetes with raised HbA1c (5.7–6.4%) and diabetes mellitus (DM).

**Results:**

Plaques in patients with raised HbA1c had a longer lipid length (17.0 ± 8.3 mm vs. 13.9 ± 7.2 mm, P = 0.004) and greater lipid index (2775.0 ± 1694.0 mm° vs. 1592.1 ± 981.2 mm°, P = 0.001) than those with normal HbA1c but were similar to DM. The prevalence of calcification in patients with raised HbA1c was significantly higher (38.7% vs. 26.3%, P = 0.048) than normal HbA1c but was similar to DM. The percentage of macrophage infiltration in the DM group was higher than that in the normal HbA1c group (20.5% vs. 7.4%, P = 0.005).

**Conclusions:**

Compared to patients with normal HbA1c, the non-culprit plaques in ACS patients with raised HbA1c had more typical vulnerable features but were similar to DM.

## Background

Diabetes mellitus (DM) and pre-diabetes mellitus (pre-DM) are common among patients with coronary artery disease (CAD) [1]. Pre-DM is a risk factor for future diabetes and cardiovascular disease, which is associated with increased risk of major adverse cardiovascular events (MACEs) [2]. According to the diagnostic criteria of American Diabetes Association (ADA), raised hemoglobinA1c (HbA1c) is defined as a glycosylated hemoglobin (Hb) value that varies from 5.7 to 6.4%, which integrates plasma glucose over time and is promoted as an indicator of pre-DM [3].

In the PROSPECT (Providing Regional Observations to Study Predictors of Events in the Coronary Tree) study, plaque burden > 70%, minimal lumen area (MLA) < 4 mm^2^, and thin-cap fibroatheroma (TCFA) are the triad of predictors of non-culprit lesion-related future MACEs [4]. A recent intravascular optical coherence tomography (OCT) study reported that the presence of lipid-rich plaques in non-culprit regions increases risk for future MACEs [5]. However, the plaque morphology in acute coronary syndrome (ACS) patients with pre-DM has not been fully illustrated. In this study, we sought to evaluate the coronary plaque morphology in patients with pre-DM by using OCT.

## Methods

### Patient population

**Fig. 1:**
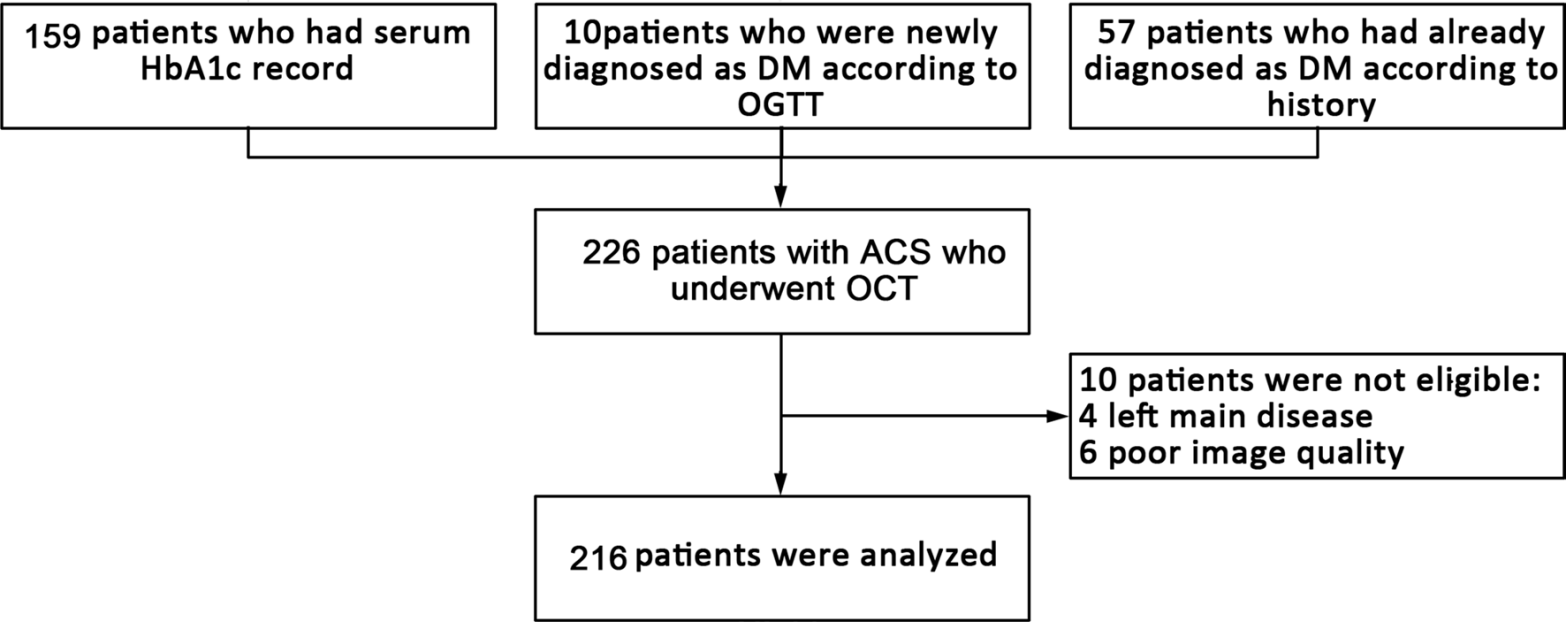
Flow chart of patients we analyzed in the present study. *ACS* acute coronary syndrome, *DM* diabetes mellitus, *HbA1c* hemoglobinA1c, *OCT* optical coherence tomography, *OGTT* oral glucose tolerance test

Study patients were retrospectively selected from the 2nd Affiliated Hospital of Harbin Medical University. Patients (aged ≥ 18 years) presenting with ACS and undergoing emergency procedures were screened for OCT examination. The main exclusion criteria were cardiogenic shock, end-stage renal disease, serious liver dysfunction, allergy to contrast media, and contraindication to aspirin or ticagrelor. Patients with left main disease, chronic total occlusion, or extremely tortuous or heavily calcified vessels were not included because of the potential difficulty in performing OCT in such situations. Clinical data including DM history, results of 75 g oral glucose tolerance test (OGTT) and HbA1c were recorded. In this study, 226 patients with ACS who underwent OCT imaging were included. Among these, 159 patients had serum HbA1c records, and 67 patients were diagnosed as DM according to OGTT (n = 10) or DM history (n = 57). Patients with poor image quality (n = 6) and left main disease (n = 4) were excluded. Finally, 216 patients were included in the analysis. The study flow chart is shown in Fig. 1. Non-culprit lesions were defined as plaques with angiographic diameter stenosis between 30 and 70% that had not been treated during the session of percutaneous coronary intervention (PCI) according to the results of stress test or ECG changes during the spontaneous ischemia attacks.

Patients were divided into three groups according to the serum level of HbA1c. Glucose metabolism was assessed according to ADA and World Health Organization (WHO) criteria [3].

Normal HbA1c (NA1c) was defined as plasma HbA1c value < 5.7%. Raised HbA1c (RA1c) was defined as plasma HbA1c value ≥ 5.7 but < 6.5% [3]. DM was defined as a history of DM, HbA1c value ≥ 6.5%, and fasting plasma glucose (FPG) level ≥ 126 mg/dL or 2-h plasma glucose (PG) level ≥ 200 mg/dL during OGTT [3]. All laboratory data were measured at fasting during the hospitalization. This study was approved by the Ethics Committee of the 2nd Affiliated Hospital of Harbin Medical University (Harbin, China), and all patients provided written informed consent.

### Angiographic analysis

Coronary angiography was performed after intra-coronary administration of 100–200 µg nitroglycerin. Quantitative coronary angiography (QCA) analysis was performed using Cardiovascular Angiography Analysis System (CAAS, 5.10, Pie Medical Imaging B.V., Maastricht, Netherlands). After selection of end-diastolic frames and calibration using the catheter’s tip, reference vessel diameter (RVD), minimal lumen diameter (MLD), diameter stenosis, and lesion length were measured.

### OCT imaging and analysis

A commercially available frequency-domain OCT system (OCT C7-XR Dragonfly, St. Jude Medical, St. Paul, MN) was used in the present study. With the help of a 6 or 7-F guiding catheter, an OCT imaging catheter was advanced distal to the lesion, and automated pullback was initiated in concordance with blood clearance by the injection of contrast media or low molecular dextran from the guiding catheter, and the OCT wire was pulled back at a rate of 20 mm/s. All images were de-identified and digitally stored.

Plaques were classified as fibrous (homogeneous, highly backscattering region) or lipid (low signal region with diffuse border). When lipid was present ≥ 90° in any of the cross-sectional images within the plaque, it was considered a lipid-rich plaque. The lipid arc was measured at every 1-mm interval throughout the length of each lesion, and the values were averaged. Lipid length was also measured on longitudinal view. Lipid index (LI) was defined as the mean lipid arc multiplied by lipid length. The fibrous cap thickness (FCT) of a lipid-rich plaque was measured 3 times at its thinnest part and the average value was calculated. A TCFA was defined as the thinnest fibrous cap with a thickness ≤ 65 μm in a lipid-rich plaque on cross-sectional imaging [6]. Each plaque was separated by at least 5 mm from the edge of another plaque or an implanted stent edge.

Calcification was an area, which consisted of a signal-poor or heterogeneous region with a sharply delineated border [7]. The number and longitudinal length of individual calcium deposits were recorded. Quantitative analysis was performed using cross-sectional OCT images at 1-mm intervals. Calcium deposits were analyzed individually by measuring calcium arc and depth (minimum distance from lumen to superficial calcium edge). Calcium index was calculated for each calcium deposit as the product of calcium length and mean calcium arc. Total calcium index was calculated as the sum of the calcium index of each calcium deposit within the whole lesion segment. The mean calcium index was calculated as the total calcium index divided by the number of calcium deposits. Spotty calcium deposits were defined as those with length < 4 mm and maximal arc < 90°, and deposits not meeting these criteria were classified as large calcium deposits.

Macrophage infiltration was defined as signal-rich, distinct or confluent punctuate regions that exceed the intensity of background spackle noise [7]. Microchannels were defined as signal-poor voids that were sharply delineated in multiple contiguous frames [8]. Plaque disruption was identified by the presence of fibrous cap discontinuity with a clear cavity formation inside the plaque.

The OCT data were analyzed by two experienced investigators who were blinded to the angiographic and clinical findings. When there was a difference between the investigators, a consensus reading was obtained from a third independent reviewer. The inter- and intra-observer kappa coefficients for lipid arc were 0.896 and 0.901, respectively.

### Statistical analysis

All statistical analyses were performed using SPSS version. 20.0 (IBM Corp, Armonk, NY, USA). Baseline clinical and imaging data were stratified on the basis of glucometabolic status. Categorical data were presented as counts (proportions) and were compared using the Chi square test or Fisher’s exact test (if the expected cell value was < 5). Continuous variables are shown as the mean ± standard deviation (SD) for normally distributed data or as median (25th–75th percentiles) for non-normally distributed data. The generalized estimating equations (GEE) methodology was used to take into account the presence of multiple plaques per patient. Between-group differences were tested using the analysis of variance (ANOVA) test. The association between HbA1c and lipid arc and lipid length was assessed using a multivariable linear analysis. All the variables with P < 0.1 in univariable analysis were entered en bloc in the multivariable model. A two-tailed *P* value < 0.05 was considered significant.

## Results

### Baseline characteristics

Baseline characteristics are presented in Table 1. The prevalence of hypertension was significantly higher in the DM group than in the other two groups (P = 0.046). The frequency of dyslipidemia was significantly higher in the RA1c and DM groups than that in the NA1c group (P = 0.021). The creatinine level and the prevalence of chronic kidney disease (CKD) were comparable among the three groups. There was no difference in statin treatment among the three groups. In the DM group, 7 subjects (8.9%) were treated with insulin therapy, and 11 subjects (13.9%) were treated with oral agents.

**Table 1:** Baseline patients characteristics

	NA1c (n = 68)	RA1c (n = 69)	DM (n = 79)	P value
Age, years	58.4 ± 9.9	59.3 ± 9.3	59.2 ± 9.2	0.849
BMI, kg/m^2^	25.1 ± 4.7	26.3 ± 3.6	26.5 ± 4.5	0.343
Male sex, n (%)	55 (80.9)	53 (76.8)	64 (81.0)	0.780
Smoking, n (%)	48 (70.6)	42 (60.9)	50 (63.3)	0.462
Hypertension, n (%)	24 (35.3)	31 (44.9)	44 (55.7)	0.046*
Dyslipidemia, n (%)	29 (42.6)	45 (65.2)	47 (59.5)	0.021*
Statin, n (%)	12 (17.6)	17 (24.6)	19 (24.1)	0.546
Prior MI, n (%)	12 (17.6)	19 (27.5)	17 (21.5)	0.373
Prior PCI, n (%)	11 (16.2)	15 (21.7)	16 (20.3)	0.695
Chronic kidney disease, n (%)	3 (4.4)	2 (2.9)	3 (3.8)	0.895
STEMI, n (%)	36 (52.9)	40 (58.0)	44 (55.7)	0.839
CK-MB, μg/L	27.7 ± 54.0	42.0 ± 116.4	42.4 ± 130.0	0.652
TnI, μg/L	7.1 ± 21.7	36.9 ± 120.7	22.1 ± 66.9	0.098
Hs-CRP, mg/dL	5.2 ± 4.8	6.4 ± 5.3	5.0 ± 5.1	0.264
Creatinine, μmol/L	76.9 ± 20.9	78.1 ± 19.8	78.4 ± 22.2	0.899
TC, mg/dL	157.3 ± 37.0	172.1 ± 47.5	164.5 ± 45.8	0.195
TG, mg/dL	117.5 ± 47.4	159.1 ± 94.0	146.5 ± 84.9	0.019
LDL-C, mg/dL	100.6 ± 35.4	112.8 ± 42.3	106.6 ± 39.6	0.248
HDL-C, mg/dL	48.2 ± 10.7	46.5 ± 10.4	47.5 ± 10.0	0.653
Insulin, n (%)	0	0	7	
Oral hypoglycemic agents, n (%)	0	0	11	

*ACS* acute coronary syndrome, *BMI* body mass index, *DM* diabetes mellitus, *MI* myocardial infarction, *NA1c* normal haemoglobinA1c, *RA1c* raised haemoglobinA1c, *PCI* percutaneous coronary intervention, *LDL-C* low-density lipoprotein cholesterol, *HDL-C* high-density lipoprotein cholesterol, *hs-CRP* high-sensitivity C-reactive protein, *STEMI* ST-segment elevation myocardial infarction, *TC* total cholesterol, *TG* triglyceride, *UA* unstable angina

Values are mean ± SD or n (%). *P < 0.05

### Angiographic findings

Angiographic findings are shown in Table 2. A total of 305 non-culprit plaques were detected in 216 subjects (95 plaques in 68 NA1c subjects, 93 plaques in 69 RA1c subjects, and 117 plaques in 79 DM subjects). The plaque distribution was not significantly different among the three groups (P = 0.966). Plaques in patients with RA1c and DM had a longer lesion length (P = 0.008). Patients with RA1c had a larger stenosis diameter percentage than the other two groups (P = 0.020), but the difference was slight.

**Table 2:** Clinical characteristics of the study population on angiography

	NA1c (n = 68)	RA1c (n = 69)	DM (n = 79)	P value	P_NA1c vs. RA1c_	P_NA1c vs. DM_	P_RA1c vs. DM_
Plaques, n	95	93	117				
Plaques/person, n	1.4 ± 0.7	1.4 ± 0.6	1.4 ± 0.7	0.473	N/A	N/A	N/A
MLA, mm	2.04 ± 0.44	1.96 ± 0.33	1.98 ± 0.42	0.370	N/A	N/A	N/A
Reference diameter, mm	3.10 ± 0.64	3.02 ± 0.53	3.00 ± 0.62	0.704	N/A	N/A	N/A
%DS	34.3 ± 2.5	35.0 ± 4.00	34.0 ± 1.3	0.020*	0.070*	0.380	0.006*
Lipid length, mm	13.9 ± 7.2	17.0 ± 8.3	16.3 ± 7.8	0.008*	0.006*	0.023*	0.516
Location				0.966	N/A	N/A	N/A
RCA							
Proximal	12 (12.6)	8 (8.6)	11 (9.4)				
Mid	9 (9.5)	10 (10.8)	8 (6.8)				
Distal	8 (8.4)	8 (10.8)	12 (10.3)				
LAD							
Proximal	7 (7.4)	14 (15.1)	12 (10.3)				
Mid	16 (16.8)	14 (15.1)	22 (18.8)				
Distal	11 (11.6)	9 (9.7)	15 (12.8)				
LCX							
Proximal	23 (24.2)	20 (21.5)	24 (20.5)				
Distal	9 (9.5)	10 (10.8)	13 (11.1)				

*DM* diabetes mellitus, *NA1c* normal haemoglobinA1c, *RA1c* raised haemoglobinA1c, *LAD* left anterior descending artery, *LCX* left circumflex artery, *RCA* right coronary artery, *%DS* percent diameter stenosis

Values are mean ± SD or n (%). *P < 0.05

### OCT findings

**Fig. 2:**
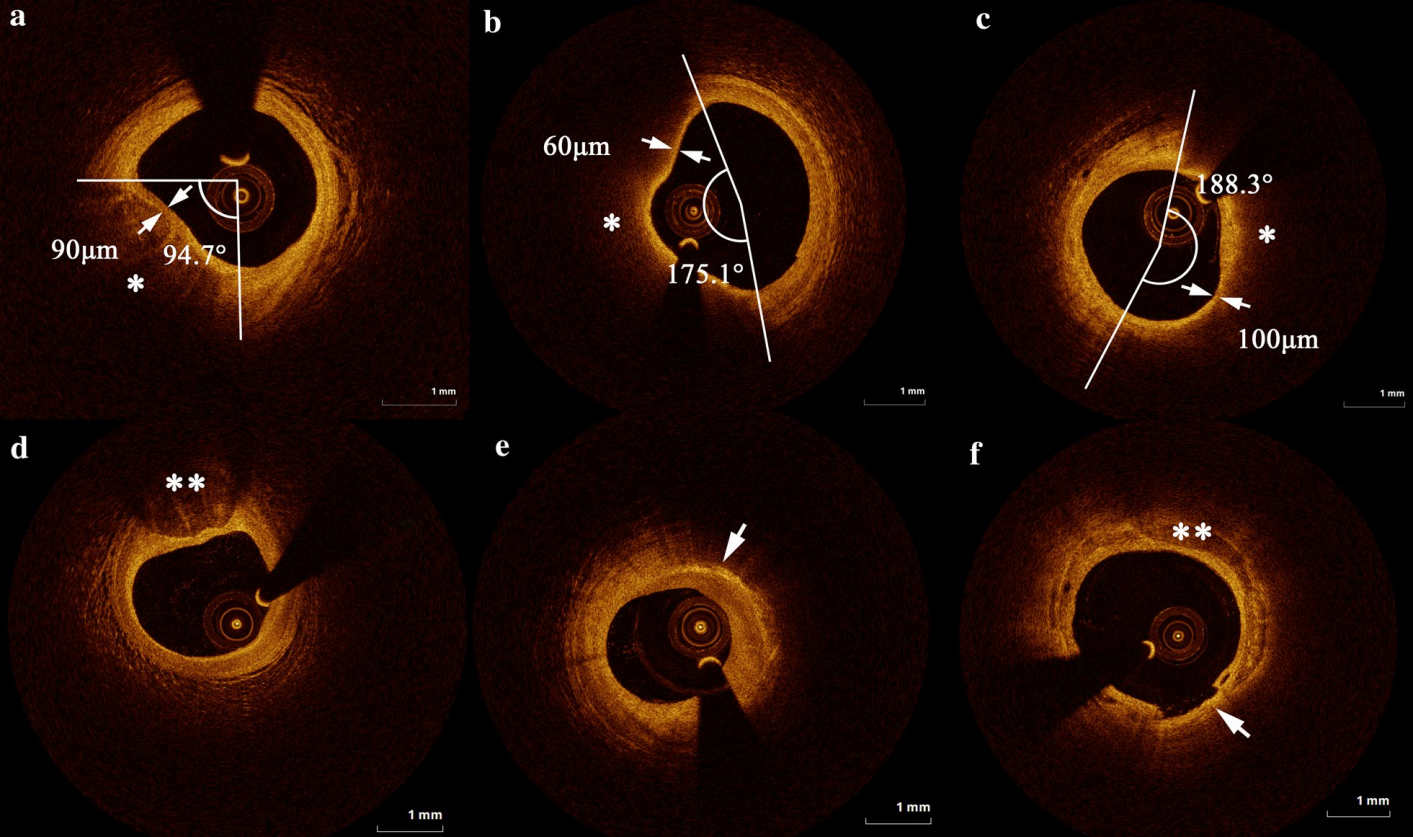
Representative cross-sectional OCT images. **a**, **b** and **c** Demonstrate lipid core and minimal fibrous cap thickness of patients with the NA1c, RA1c and DM, respectively. The arc of the lipid core was characterized by a signal-poor region and a diffuse border was measured, the thinnest part (arrows) of the fibrous cap identified as a signal-rich homogenous region overlying a lipid core (*) was measured. **d** The calcification (**) present in a patient with RA1c. **e** The macrophages in a patient with DM. **f** The calcification (**) in a patient with DM, disruption can also be seen (arrow). *DM* diabetes mellitus, *NA1c* normal hemoglobinA1c, *RA1c* raised hemoglobinA1c

**Fig. 3:**
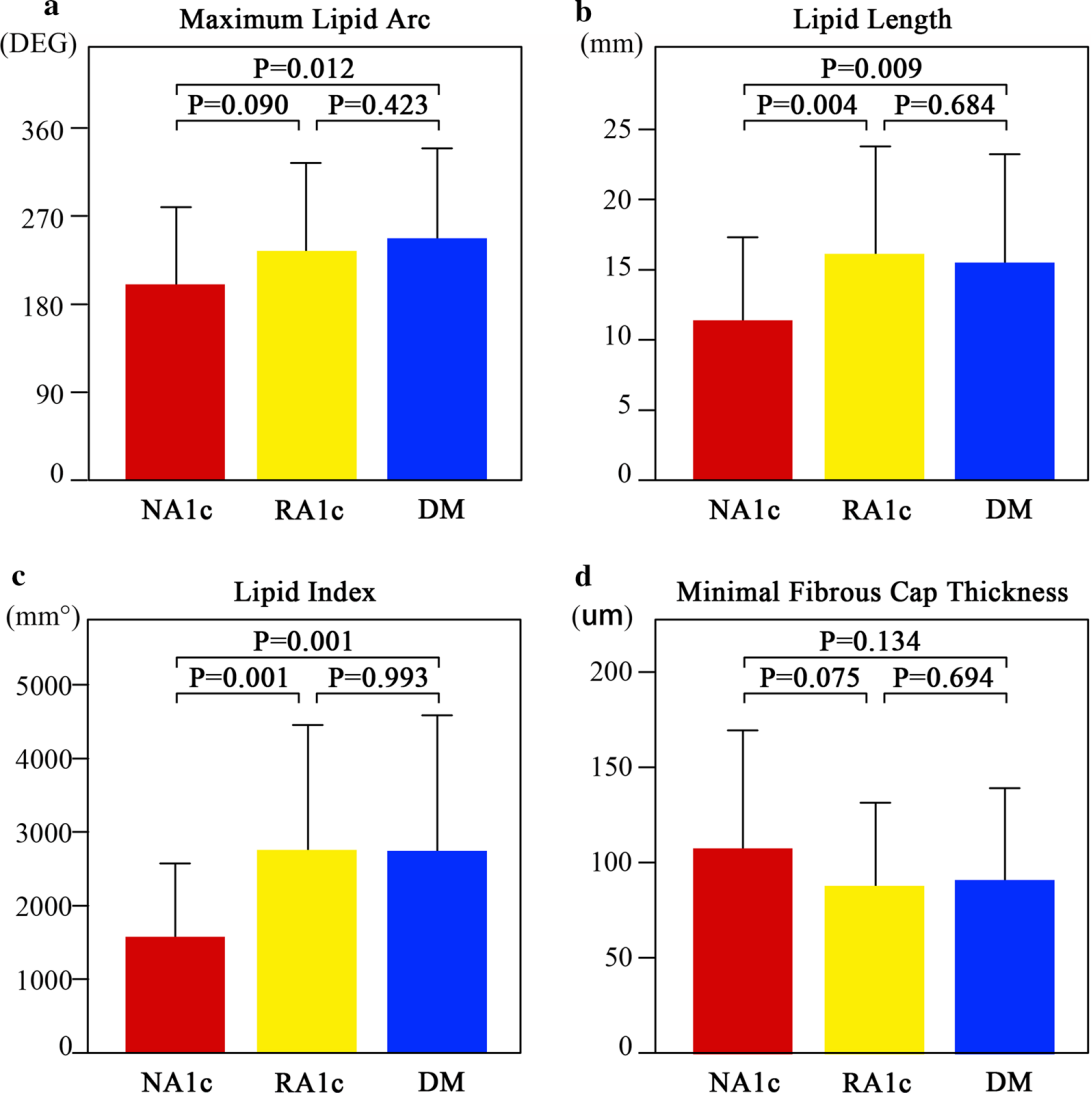
Comparisons of OCT findings of coronary plaques containing lipid core between NA1c, RA1c, and DM groups. Maximum lipid arc (**a**), lipid length (**b**), lipid index (**c**) and minimal fibrous cap thickness (**d**). Data are expressed as the mean ± SD. *DM* diabetes mellitus, *NA1c* normal hemoglobinA1c, *RA1c* raised hemoglobinA1c

Representative OCT images in each group are shown in Fig. 2. A comparison of the quantitative OCT findings of the lipid plaques between the NA1c, RA1c, and DM groups is presented in Fig. 3. The plaques had a significantly longer lipid length and greater LI in the RA1c and DM groups than in the NA1c group (lipid length: 17.0 ± 8.3 mm [RA1c] vs. 13.9 ± 7.2 mm [NA1c], P = 0.004 and 16.3 ± 7.8 mm [DM] vs. 13.9 ± 7.2 mm [NA1c], P = 0.009; LI: 2755.0 ± 1694.0 mm° [RA1c] vs. 1592.1 ± 981.2 mm° [NA1c], P = 0.001 and 2758.0 ± 1821.7 mm° [DM] vs. 1592.1 ± 981.2 mm° [NA1c], P = 0.001). Compared to patients with NA1c, plaques in those with DM had a significantly wider maximum lipid arc and mean lipid arc (maximum lipid arc: 246.8 ± 90.6° [DM] vs. 199.7 ± 79.9° [NA1c], P = 0.012; mean lipid arc: 175.2 ± 63.7° [DM] vs. 142.2 ± 57.1° [NA1c], P = 0.012). Additionally, the maximum and mean lipid arcs were wider in patients with RA1c than in those with NA1c with a borderline significance (maximum lipid arc: 232.4 ± 91.3° [RA1c] vs. 199.7 ± 79.9° [NA1c], P = 0.090; mean lipid arc: 164.7 ± 63.7° [RA1c] vs. 142.2 ± 57.1° [NA1c], P = 0.090). There were no significant differences in these lipid plaque parameters between the RA1c and DM groups.

The FCT of plaques in RA1c subjects and DM subjects tended to be thinner than that in NA1c subjects, although there was no significant difference (107.2 ± 61.9 mm [NA1c] vs. 87.0 ± 44.2 mm [RA1c] vs. 91.1 ± 47.9 mm [DM], P = 0.167).

In univariable linear regression (Table 3), HbA1c was significantly correlated with maximal lipid arc (coefficient β = 15.469, P = 0.002) and lipid length (coefficient β = 1.678, P < 0.001). All the variables with P < 0.1 were entered en bloc in the multivariable model. As result, the model of maximal lipid arc was adjusted by age, smoking, HbA1c, triglyceride (TG) and creatinine, and the model of lipid length was adjusted by HbA1c, TG and high-density lipoprotein cholesterol (HDL-C). In the multivariable linear regression analysis (Table 4), a higher HbA1c was independently associated with a larger maximal lipid arc (coefficient β = 14.282, P = 0.005) and a longer lipid length (coefficient β = 1.368, P = 0.003).

**Table 3:** Univariable linear regression

Variable	Maximal lipid arc		Lipid length	
	Coefficients	P value	Coefficients	P value
Age	1.731 [0.213 to 3.25]	0.026	− 0.105 [− 0.241 to 0.031]	0.801
Male	16.734 [− 19.134 to 52.603]	0.358	0.711 [− 2.476 to 3.898]	0.457
BMI	− 0.393 [− 6.995 to 6.21]	0.905	0.202 [− 0.284 to 0.688]	0.507
Smoking	25.688 [− 3.812 to 55.189]	0.087	1.603 [− 1.025 to 4.231]	0.041
Hypertension	8.539 [− 19.310 to 36.388]	0.546	1.755 [− 0.702 to 4.211]	0.069
Dyslipidemia	10.902 [− 16.926 to 38.730]	0.440	2.394 [− 0.048 to 4.837]	0.105
HbA1c	15.469 [5.965 to 24.982]	0.002	1.678 [0.848 to 2.507]	< 0.001
TC	0.148 [− 0.218 to 0.514]	0.426	0.025 [− 0.006 to 0.057]	0.069
TG	0.221 [0.066 to 0.376]	0.006	0.014 [0 to 0.028]	0.001
HDL-C	− 0.537 [− 1.785 to 0.712]	0.396	− 0.147 [− 0.253 to − 0.04]	0.011
LDL-C	− 0.003 [− 0.406 to 0.401]	0.989	0.026 [− 0.010 to 0.061]	0.244
Creatinine	0.720 [0.087 to 1.354]	0.026	− 0.032 [− 0.089 to 0.025]	0.677
Statin	− 20.074 [− 58.734 to 18.587]	0.307	− 0.223 [− 3.662 to 3.216]	0.286
Prior MI	− 18.604 [− 52.728 to 15.520]	0.283	1.686 [− 1.339 to 4.711]	0.863
CKD	− 18.982 [− 78.979 to 41.014]	0.533	− 8.765 [− 13.908 to − 3.622]	0.300
STEMI	22.339 [− 6.329 to 51.007]	0.126	− 1.767 [− 4.312 to 0.779]	0.892
Hs-CRP	0.382 [− 2.66 to 3.423]	0.804	− 0.287 [− 0.548 to − 0.025]	0.707

*BMI* body mass index, *CKD* chronic kidney disease, *LDL-C* low-density lipoprotein cholesterol, *HDL-C* high-density lipoprotein cholesterol, *HbA1c* hemoglobinA1c, *TC* total cholesterol, *TG* triglyceride

**Table 4:** Multivariable linear regression

Variable	Coefficients	P value
For maximal lipid arc		
Age	1.933 [0.311 to 3.554]	0.020
Smoking	48.34 [17.931 to 78.749]	0.002
HbA1c	14.282 [4.417 to 24.147]	0.005
TG	0.174 [0.024 to 0.323]	0.023
Creatinine	0.427 [− 0.217 to 1.071]	0.192
For lipid length		
HbA1c	1.368 [0.469 to 2.267]	0.003
TG	0.005 [− 0.009 to 0.019]	0.487
HDL-C	− 0.114 [− 0.223 to − 0.004]	0.042

*HDL-C* high-density lipoprotein cholesterol, *HbA1c* hemoglobinA1c, *TC* total cholesterol, *TG* triglyceride

The prevalence of OCT plaque characteristics are shown in Table 5. Of the 305 non-culprit plaques, 157 were found to contain a lipid core (40, 43 and 54 in the NA1c, RA1c and DM groups, respectively). The prevalence of lipid-rich plaques was similar among NA1c, RA1c and DM groups (P = 0.735).

**Table 5:** Plaque characteristics in non-culprit lesion on optical coherence tomography

	NA1c (n = 95)	RA1c (n = 93)	DM (n = 117)	P value	P_NA1c vs. RA1c_	P_NA1c vs. DM_	P_NA1c vs. DM_
Lipid, n (%)	40 (42.1)	43 (46.2)	54 (46.2)	0.802	N/A	N/A	N/A
Lipid-rich plaques, n (%)	38 (40.0)	42 (45.2)	52 (44.4)	0.735	N/A	N/A	N/A
TCFA, n (%)	12 (12.6)	19 (20.4)	18 (15.4)	0.335	N/A	N/A	N/A
Disruption, n (%)	1 (1.1)	2 (2.2)	1 (0.9)	0.689	N/A	N/A	N/A
Crystal, n (%)	4 (4.2)	0 (0.0)	6 (5.1)	0.097	N/A	N/A	N/A
Macrophage, n (%)	7 (7.4)	11 (11.8)	24 (20.5)	0.018*	0.215	0.005*	0.067
Microchannels, n (%)	7 (7.4)	9 (9.7)	15 (12.8)	0.419	N/A	N/A	N/A
Thrombus presence, n (%)	2 (2.1)	2 (2.2)	1 (0.9)	0.696	N/A	N/A	N/A
Calcification, n (%)	25 (26.3)	36 (38.7)	55 (47.0)	0.008*	0.048*	0.001*	0.143
Spotty calcification, n (%)	19 (20.0)	26 (28.0)	43 (36.8)	0.027	0.134	0.006*	0.115
Large calcification, n (%)	6 (6.3)	10 (10.8)	15 (12.8)	0.289	N/A	N/A	N/A

*DM* diabetes mellitus, *NA1c* normal haemoglobinA1c, *RA1c* raised haemoglobinA1c, *TCFA* thin-cap fibroatheroma

Values are mean ± SD or n (%). *P < 0.05

The frequency of calcification in RA1c group (P = 0.048) and DM group (P = 0.001) was higher than that in NA1c group. There was a significant difference in the prevalence of spotty calcification between DM group and NA1c group (36.8% vs. 20.0%, P = 0.006). Detailed OCT analysis of calcium deposits is summarized in Table 6.

**Table 6:** Optical coherence tomography calcium analysis

	NA1c (n = 25)	RA1c (n = 36)	DM (n = 55)	P value
Total calcifications/lesion	1.0 (1.0, 1.5)	1.0 (1.0, 2.0)	1.0 (1.0,2.0)	0.714
Spotty calcium/lesion	1.0 (0.5, 1.0)	1.0 (1.0, 1.0)	1.0 (1.0, 1.0)	0.969
Large calcium/lesion	0.0 (1.0, 1.0)	0.0 (0.0, 1.0)	0.0 (0.0, 1.0)	0.907
Total calcium index, mm°	417.5 ± 426.9	438.8 ± 475.9	496.4 ± 479.2	0.807
Mean calcium index, mm°	248.6 ± 207.0	261.4 ± 192.2	308.1 ± 227.2	0.538
Maximal calcium arc, °	74.8 ± 25.2	88.4 ± 34.1	95.0 ± 44.7	0.095
Mean calcium arc, °	52.9 ± 17.8	62.5 ± 24.1	67.1 ± 31.6	0.095
Mean calcium length, mm	3.9 ± 3.3	4.3 ± 2.6	4.6 ± 3.6	0.662
Mini calcium depth, μm	151.6 ± 117.0	132.5 ± 97.9	139.8 ± 116.1	0.804

*DM* diabetes mellitus, *NA1c* normal haemoglobinA1c, *RA1c* raised haemoglobinA1c

Values are mean ± SD or median (interquartile range). *P < 0.05

The prevalence of TCFA (P = 0.335), disruption (P = 0.689), thrombus (P = 0.696) and microchannels (P = 0.419) was not different among the three groups. The prevalence of macrophage infiltration in the DM group was higher than that in the NA1c group (P = 0.005). Although the frequency of macrophage infiltration tended to be higher in RA1c subjects than in NA1c subjects, the difference was not significant (P = 0.215).

## Discussion

This study aimed to investigate the morphologic characteristics of non-culprit plaques in ACS patients with RA1c. The lipid core of plaques was larger and calcification was more common in patients with RA1c than in patients with NA1c, similar to those with DM. DM patients had a higher prevalence of macrophages.

Due to high resolution (10–15 μm), OCT offers a more accurate and reliable depiction of specific plaque components in vivo, particularly microstructural vulnerable plaque features such as the FCT and the presence of macrophages, which has been established superior to other conventional imaging techniques such as intravascular ultra sound [9]. Previous OCT studies revealed the impairment of DM to plaque vulnerability. Reith et al. [10] found the frequency of TCFA was higher in diabetic patients compared to non-diabetic patients. Patients with DM were found, had a lower minimal FCT of the coronary target lesion than those with non-DM [11, 12]. Several studies found that patients with DM had a larger LI suggesting a larger lipid core, compared with non-DM patients [12, 13]. Kuroda et al. [14] reported that glucose fluctuation contributed to the formation of lipid-rich plaques and thinning the FCT. Consistently, the present study found a greater size of lipid core in patients with DM. Notably, we did not find a significant difference in FCT and prevalence of TCFA among patients with NA1c, RA1c or DM. Feng et al. [15] found the FCT and the frequency of TCFA were comparable between patients with DM and non-DM. Recently, Xing et al. [5] reported that the presence of lipid-rich plaque, but not the TCFA and the FCT assessed by OCT, was correlated with increased risk of non-culprit lesion-related MACEs.

Suzuki et al. [12] found that CAD patients with impaired glucose tolerance (IGT) have a greater size of lipid core and thinner FCT than those with normal glucose tolerance. A postmortem study showed a positive correlation between mean percent necrotic core size and the HbA1c level [16]. An OCT study found a significant direct correlation between HbA1c and prevalence of lipid plaques and TCFA [17]. The present study found patients with RA1c had a lager lipid core than with NA1c, similar to with DM. We also found the correlation of HbA1c with lipid size. The present study assessed exclusively ACS patients and diagnosed pre-DM as raised HbA1c, which differ from the study by Suzuki [12].

Previous physiological studies reported that long-term exposure to hyperglycemia and excess free fatty acids, together with insulin resistance in DM, resulted in dysfunction of endothelial cells, which augments vasoconstriction, increases inflammation, and promotes thrombosis, thereby promoting coronary atherosclerosis [18]. This process may initiate in the pre-diabetes phase. An animal study revealed that pre-DM increased oxidative stress [19]. CKD was reported another cause of systemic inflammation and oxidative stress and was recognized as an independent risk factor for CAD [20]. All these studies suggested that the function of endothelial cells may be impaired and advanced atherosclerotic plaque may form in patients with RA1c.

Coronary artery calcification is well associated with advanced atherosclerosis and increased risk of adverse cardiac events [21]. Therefore, subjects with coronary artery calcium have a higher short-term risk of death [22]. Previous studies showed that the prevalence of calcified plaques in the DM group was significantly higher than that in the non-DM group [17, 23, 24]. In the present study, we found that the RA1c and DM groups had a higher prevalence of calcified plaques, which was consistent with previous studies. Additionally, several studies found that spotty and superficial calcium deposits were frequently in culprit lesion of patients with ACS, suggesting an association with plaque vulnerability [25–27]. However, a recent OCT study did not find any difference between patients with myocardial infarction and stable pectoris in the pattern of culprit plaque calcification [28]. In the latter study, calcium analysis was restricted to a shorter culprit segment (10 mm-long). Niccoli et al. [29] found that superficial calcified nodules were more frequently found in diabetic than in non-diabetic patients. The present study found that the frequency of spotty calcification was higher in DM group than in NA1c group.

Pathologic study revealed coronary tissue from patients with DM exhibited a larger content of macrophage infiltration than tissue from patients without DM [30]. Mita et al. [31] demonstrated that oscillations in blood glucose concentrations accelerated macrophage adhesion to the endothelial surface. An increased number of macrophages was related to necrotic core expansion and plaque instability [16]. Additionally, high glucose concentration increased the inflammatory activity of macrophages [32]. In this study, there was a higher prevalence of macrophage infiltration in patients with DM, which is consistent with previous studies [17]. Additionally, the prevalence of macrophage infiltration in patients with RA1c was higher than those with NA1c, although the difference was not significant.

Previous studies reported that HbA1c was significantly associated with ischemic heart disease mortality [33, 34]. Additionally, HbA1c has the advantage of wider application, in that it can be performed regardless of fasting or timed samples, is largely unaffected by acute illness, and may be used to guide management and to adjust therapies [35]. Taken together with the findings of the present study, the importance of HbA1c is enhanced in CAD patients, which will allow the timely diagnosis of pre-DM or latent DM.

The results of the present study indicated that an abnormality in glucose metabolism should be managed as early as possible in the prevention of cardiovascular disease [36]. The preferred approach for this is intensive lifestyle intervention, which besides reducing progression to diabetes, has also been shown to reduce all-cause mortality in a long-term follow-up study [37]. The best evidence for a pharmacological approach is with metformin [38]. However, a large-scale prospective study is warranted to evaluate whether additional glucose control will decrease the progression of plaque vulnerability and late clinical events.

### Study limitations

In the present study, there were several limitations. First, this was a retrospective study using a registry database. Therefore, potential selection bias is unavoidable. Patients with cardiogenic shock (hemodynamic instability), congestive heart failure, chronic total occlusion, left main disease, or renal failure were not included. Second, FPG and 2-h PG concentrations may be increased in ACS, which increases the risk of inadvertently classifying patients with non-DM as having DM. Third, the exact measurements of necrotic core and plaque burden by OCT were not possible because of the relatively shallow axial penetration. However, because the most important morphological determinants of plaque vulnerability are superficial, the region of greatest interest was still within the imaging range of current OCT systems. Finally, the impairment of duration of RA1c to plaque vulnerability was not considered in this study.

## Conclusions

Compared to patients with NA1c, the non-culprit plaques in ACS patients with RA1c were more vulnerable, similar to DM. Thus, preventing patients from progressing from pre-DM to DM is important.

## Abbreviations

ACS: acute coronary syndrome
ADA: American Diabetes Association
CAD: coronary artery disease
CKD: chronic kidney disease
DM: diabetes mellitus
FCT: fibrous cap thickness
FPG: fasting plasma glucose
HbA1c: hemoglobinA1c
IFG: impaired fasting glucose
IGT: impaired glucose tolerance
LI: lipid index
MACEs: major adverse cardiovascular events
MLA: minimal lumen area
MLD: minimal lumen diameter
NA1c: normal hemoglobinA1c
OCT: optical coherence tomography
OGTT: oral glucose tolerance test
PG: plasma glucose
pre-DM: pre-diabetes mellitus
QCA: quantitative coronary angiography
RA1c: raised hemoglobinA1c
RVD: reference vessel diameter
TCFA: thin-cap fibroatheroma
WHO: World Health Organization
